# Getting better at chronic care in remote communities: study protocol for a pragmatic cluster randomised controlled of community based management

**DOI:** 10.1186/1471-2458-12-1017

**Published:** 2012-11-21

**Authors:** Barbara Schmidt, Mark Wenitong, Adrian Esterman, Wendy Hoy, Leonie Segal, Sean Taylor, Cilla Preece, Alex Sticpewich, Robyn McDermott

**Affiliations:** 1 Getting Better at Chronic Care Project, University of South Australia, School of Health Sciences, Cairns Diabetes Centre, 381 Sheridan St, North Cairns, QLD, 4870, Australia; 2 Apunipima Cape York Health Council, 186 McCoombe St, Bungalow Cairns, QLD, 4870, Australia; 3 Sansom Institute of Health Service, School of Health Sciences, University of South Australia, Level 6, Centenary Building, North Terrace, Adelaide, 5000, Australia; 4 University of Queensland School of Medicine, Centre for Chronic Disease, Health Sciences Building, Level 8 Royal Brisbane and Womens Hospital, Herston, Brisbane, 4029, Australia; 5 School of Nursing and Midwifery, Division of Health Sciences, University of South Australia, Playford Building P4-26, City East Campus, North Terrace, Adelaide, 5000, S.A, Australia; 6 Sansom Institute of Health Service, School of Health Sciences, University of South Australia, University of South Australia City East Campus North Terrace, Adelaide, 5000, S.A, Australia

**Keywords:** Aboriginal and Torres Strait Islander, Diabetes, Indigenous Health Worker, Partnerships, HbA1c control

## Abstract

**Background:**

Prevalence and incidence of diabetes and other common comorbid conditions (hypertension, coronary heart disease, renal disease and chronic lung disease) are extremely high among Indigenous Australians. Recent measures to improve quality of preventive care in Indigenous community settings, while apparently successful at increasing screening and routine check-up rates, have shown only modest or little improvements in appropriate care such as the introduction of insulin and other scaled-up drug regimens in line with evidence-based guidelines, together with support for risk factor reduction. A new strategy is required to ensure high quality integrated family-centred care is available locally, with continuity and cultural safety, by community-based care coordinators with appropriate system supports.

**Methods/design:**

The trial design is open parallel cluster randomised controlled trial. The objective of this pragmatic trial is to test the effectiveness of a model of health service delivery that facilitates integrated community-based, intensive chronic condition management, compared with usual care, in rural and remote Indigenous primary health care services in north Queensland. Participants are Indigenous adults (aged 18–65 years) with poorly controlled diabetes (HbA1c>=8.5) and at least one other chronic condition. The intervention is to employ an Indigenous Health Worker to case manage the care of a maximum caseload of 30 participants. The Indigenous Health Workers receive intensive clinical training initially, and throughout the study, to ensure they are competent to coordinate care for people with chronic conditions. The Indigenous Health Workers, supported by the local primary health care (PHC) team and an Indigenous Clinical Support Team, will manage care, including coordinating access to multidisciplinary team care based on best practice standards. Allocation by cluster to the intervention and control groups is by simple randomisation after participant enrolment. Participants in the control group will receive usual care, and will be wait-listed to receive a revised model of the intervention informed by the data analysis. The primary outcome is reduction in HbA1c measured at 18 months. Implementation fidelity will be monitored and a qualitative investigation (methods to be determined) will aim to identify elements of the model which may influence health outcomes for Indigenous people with chronic conditions.

**Discussion:**

This pragmatic trial will test a culturally-sound family-centred model of care with supported case management by IHWs to improve outcomes for people with complex chronic care needs. This trial is now in the intervention phase.

**Trial registration:**

Australian New Zealand Clinical Trials Registry ACTR12610000812099

## Background

Prevalence and incidence of Type 2 diabetes mellitus (T2DM) and other common comorbid conditions (hypertension, coronary heart disease (CHD), chronic kidney disease (CKD) and chronic obstructive pulmonary disease (COPD) are extremely high among Indigenous Australians. Cardiovascular and other complications which can be prevented with good quality primary care remain 4–7 times higher than for the general population [1]. This reflects lower access to appropriate and effective preventive care, as well as ongoing poor nutrition, life-course exposures and high rates of tobacco exposure [1]. Vos and colleagues [1] estimate the Indigenous life expectancy gap at 13 years, and 59% of the total burden of disease as preventable. Most of this excess is due to chronic conditions in adults, mainly cardiovascular disease (CVD), diabetes, mental disorders and chronic lung disease [1]. Rates for preventable hospitalisations are more than double in remote and very remote communities compared with major cities [2].

There is strong evidence, including from the United Kingdom Prospective Diabetes Study (UKPDS) [3], that the main goal in treating T2DM should be to achieve blood glucose levels as close as possible to the non-diabetic range to prevent chronic microvascular and macrovascular complications, especially nephropathy. This evidence is recognised by key national bodies, such as the Australian Diabetes Society [4] and the American Diabetes Association [5], which recommend a glycated haemoglobin (HbA1c) target of <7.0% for most adults with diabetes. The progressive nature of T2DM means that many people need intensification of drug therapy over time if this target is to be achieved. For example, projections from UKPDS data indicate that most patients will need insulin therapy to maintain HbA1c <7.0% after 9 years of diagnosed T2DM [6]. In addition, the appropriate use of medicines, including angiotensin converting enzyme inhibitors (ACEI) to protect the kidneys and heart, reduces all-cause and CVD-specific mortality as well as progression to end-stage renal disease [7]. Good management of kidney disease and its attendant cardiovascular risk requires: active stepped care with renin-angiotensin system blockade (ACEI or angiotensin receptor blockers, ARB); diuretics, calcium channel blockers and other drugs to achieve blood pressure goals; and attempts to back-titrate albuminuria/proteinuria, with ACEI or ARB [8]. Good management of CVD in this population with high levels of other conditions may require five classes of drugs including beta blockers, antiplatelet agents, ACEI/ARB, statins and nitrates, as well as good support for smoking cessation, better nutrition and physical activity [8]. In many cases, current care falls well below these standards. Family engagement is critical to achieving this for individuals, and in remote Indigenous communities we believe Indigenous Health Workers (IHWs) are best placed to deliver this “package” in a way which is acceptable and understandable for clients [9].

Recent measures to improve quality of preventive care in Indigenous community settings, while apparently successful at increasing screening and routine check-up rates (mainly performed by nurses and IHWs) [10], have shown only modest or little improvements in appropriate doctor-initiated care, specifically the introduction of insulin and other scaled up drug regimens in line with evidence-based guidelines [11]. Where improvements have been demonstrated, the longer term sustainability of these has been less than hoped, due to, inter alia, failure of integration of these systems into ongoing service delivery models and especially of the up-skilling of the Indigenous workforce with appropriate system-level support [11]. A similar experience has been reported in the Indian Health Service in the USA, where “data alone are not sufficient to effect change. Use of the measures to ensure that the quality of care improves must also be stressed, because measuring alone will not guarantee such improvement” [12]. Although multidisciplinary support for chronic care is the rule in mainstream settings, this suite of allied health services is not routinely available to many rural and remote populations, nor are doctors and nurses trained to deliver these services. Effective communication between health care workers and clients is central to good quality chronic care, especially in populations with low health literacy. A study of people with diabetes with low health literacy showed that an HbA1c of <8.6% was up to nine times more likely when physicians used an interactive educational strategy assessing comprehension of new concepts [9].

A trial of health worker-delivered diabetes care in the Torres Strait in 2000 demonstrated that much of this type of care could be effectively given in the community by local Indigenous health workers who were close to the client group linguistically and culturally and provided greater continuity of care than itinerant, often inexperienced non-Indigenous health staff [13]. Follow-up after three years showed sustained improvements in service delivery and intermediate clinical outcomes (blood pressure, glycaemia and complications of diabetes) demonstrating that system-level support for this model could produce ongoing clinical gains [14] and also be highly cost-effective [15].

Similarly, a treatment program to modify renal and cardiovascular disease progression in an Aboriginal community in the Northern Territory (NT), which concentrated on improved drug management of hypertension and renal disease, showed statistically significant protection against dialysis and natural death over a 3 year period [16] and also demonstrated high cost-effectiveness [17]. An outreach program in the NT and Western Australia showed an increase in prescribed measurements when screening was regularised and decision algorithms were established and supported [18]. These programs and others show that Indigenous people can participate enthusiastically in chronic condition management, with improvement in clinical profiles, preventable hospitalisations and mortality.

It is our view that Indigenous health care workers in many rural and remote communities remain underutilised in chronic care, especially where cultural safety and dialogue with the extended family is key to better medium- and long-term management. This presents an opportunity to improve health care standards in high need populations. A recent review of progress in diabetes and renal care in the Torres Strait showed that, while clinical registers showed a doubling of the diabetes caseload in five years, there was little shift in intermediate clinical indicators [19], reflecting a stalling in doctor-initiated care, including insulin management and self-monitoring of blood glucose. At the same time, numbers of clients on renal care plans have increased markedly, potentially overwhelming existing dialysis services in the region [20].

Great disparities remain between reported glycaemia in Indigenous people compared with the general Australian population of people with diabetes, where 38% achieved an HbA1c level of less than 7% compared to 26% or less among Indigenous adults with diabetes [19]. This was reflected in low rates of self-monitoring (4% vs 58%) and insulin treatment (10.5% vs 34.4%), and very different albuminuria prevalence (34.4% vs 7%) for Aboriginal and non-Aboriginal adults with diabetes respectively [21]

This problem of suboptimal primary level preventive care for chronic conditions is also documented in mainstream clinical settings, where apparent “clinical inertia” by doctors results in suboptimal blood pressure, lipid and glycaemic control. Over a decade ago the UKPDS report demonstrated that glycaemia was an important driver of microvascular complications in diabetes, and that chronic complications, especially nephropathy, could be reduced by 38% for each 1% decrease in HbA1c achieved [22]. Yet suboptimal uptake of relatively simple clinical protocols is still highly prevalent [23]. Diabetes is now the single greatest and growing cause of renal failure requiring dialysis globally. It is becoming clear that the rate of growth of the traditional professional health workforce, and the system-level support behind it, is insufficient to deal effectively with the current rise in chronic conditions.

For existing and future underserved populations, with growing needs for quality evidence-based care and with high rates of comorbid conditions, a new strategy is required. This new approach must deliver high quality integrated family-centred care locally, with continuity and cultural safety, by community-based professionals and with appropriate system support. Because of this, our approach is based on a different model of care. A “family-centred” approach has been developed in consultation with Cape York Aboriginal communities and Indigenous health workers to implement health reform in Cape York and is based on several evidence based programs. It aims to proactively address chronic conditions at both family and individual level and embed a family-centred approach into the service delivery systems approach to provide more effective and culturally safe health service delivery that is sustainable in the longer term [24]. Glasgow (2003) argues that successful translation of research into practice in chronic care requires methods that will:

1.“enhance and measure the reach of the interventions, especially toward poor, undeserved and minority populations;

2.develop programs that can be adopted in diverse settings;

3.produce replicable effects and enhance quality of life, in addition to short term behavioural or biological outcomes;

4.be consistently implemented by different staff members having moderate levels of training; and

5.produce maintenance at both individual and setting levels at reasonable cost” [25].

This trial forms part of a broader project that aims to introduce and evaluate a new strategy for integrated community-based, intensive chronic condition management in rural and remote Indigenous primary care services in north Queensland.

The project will occur in three stages over five years:

1.A randomised controlled trial of intensive locally-delivered chronic care in 12 participating sites in Far North Queensland, with clinical and quality of life outcomes. A cluster randomised control design has been used to avoid contamination bias in results that may arise from control and intervention participants being in the same community [26].

2.Review of the model in the light of the trial results, discussions about generalisability and associated process evaluation and qualitative enquiries, with development of an implementation plan (encompassing potential regional implications of a family-centred service delivery model, including workforce and funding applications) and the refinement of the project training program.

3.In collaboration with the trial partners (Queensland Health, Apunipima Cape York Health Council and local Aboriginal Medical Services) implementation of the revised model will occur in the wait listed communities.

Ethics approval of the protocol was granted in November 2010.

## Method/design

**Figure 1:**
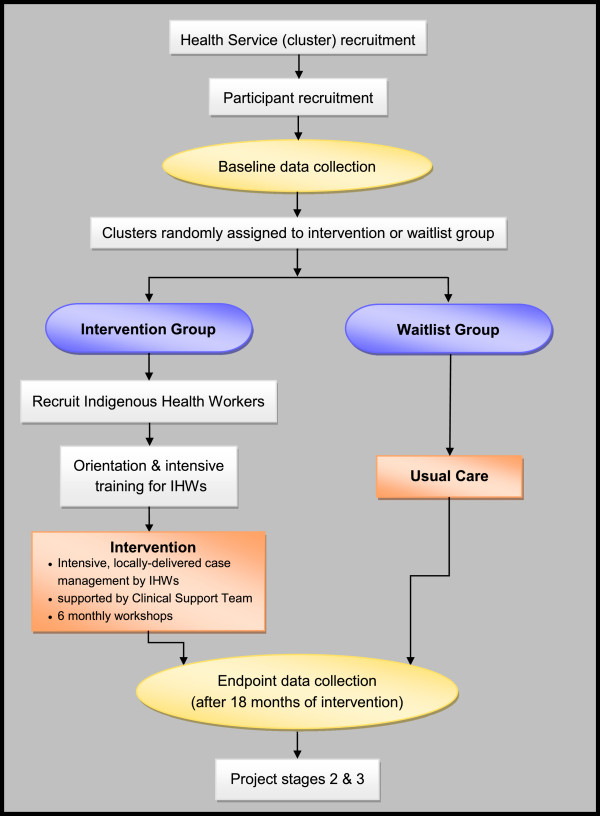
**Flowchart for a cluster randomised controlled trial of family**-**centred chronic care delivered by IHWs to Indigenous clients.**

A project flow diagram illustrating the research design is shown in Figure 1.

### Participants

The setting for the study is 12 primary health care services located in 12 rural north Queensland communities which have a significant Indigenous population, and where the service is provided either by Queensland Health or a local community-controlled Indigenous Health Service.

The project was initiated by University of South Australia in partnership with Queensland Health, Apunipima Cape York Health Council and the University of Queensland in July 2010. The decisions about which communities to approach to be involved in the study was made in consultation with the key service delivery partners. Involving the partners was a key strategy to ensure communities selected had the service capacity and organisational support to be involved in the project.

Participants in the study are Aboriginal and Torres Strait Islander people diagnosed at least one year prior to recruitment with T2DM with HbA1c ≥8.5%, plus at least one of either COPD, CHD, CKD (Stages 1 to 3), or hypertension.

The exclusion criteria are:

• People aged > 65 years;

• Children and/or young people (<18 years);

• People with an intellectual or mental impairment (people with major mental illness);

• Women who are pregnant;

• CKD Stages 4 & 5.

These participants are clustered by health service.

### Intervention

One IHW full time equivalent (FTE) who is eligible at IHW Level 004 [27] will be recruited for each cluster (maximum 30 participants) in the intervention group, The role of the IHW is to coordinate the care of participants in their community.

As this is a pragmatic trial, there are both standardised and contextualised elements of the intervention, which are listed in Table 1.

**Table 1:** Standardised and Contextualised elements of the intervention

	**Standardised elements**	**Contextualised elements**
Family-centred case management by IHW	· Qualifications of HW (min Certificate IV in Aboriginal and Torres Strait Islander Primary Health Care)	· use local PHC information systems
	· Caseload 1.0 FTE:15-30 clients	· use local referral pathways
	· IHW supernumerary to primary health care team	· use local care planning templates
	· Training & orientation program (72 hrs face-to-face): Competency-based training in primary, secondary and tertiary health promotion interventions and clinical management of diabetes and COPD, CKD (Stages 1-3), hypertension and CHD	· use local education resources
	· Supervisors attend orientation workshop	· Level and nature of contact with clients is at IHW’s discretion: ie they determine the appropriate language, resources, frequency and setting for care and education (home visits etc) according to client needs
	· 6-monthly training (one week)	
	· Chronic Disease Guidelines (2010) as clinical governance protocol	
	· COPD screening with Piko 6 spirometer	
System Support	· Trial manager	· Weekly support uses reflective practice technique, responding to the needs and context of each HW
	· 2 FTE ICST for 6 HWs	· Problem solving for local context, eg working with local team to establish/facilitate care plan process, sorting contract issues etc
	· Weekly report and plan	
	· Weekly meeting (phone or video)	
	· Remote clinical supervision of caseload	
	· Monthly IHW meeting by videoconference	

An Indigenous Clinical Support Team (ICST) that consists of a registered nurse (s) and IHW will train and mentor the community-based IHWs to coordinate intensive management for five common chronic conditions, according to the clinical and service goals outlined in the Chronic Disease Strategy [28]. Specific training and support will be provided for:

• Evidence-based management and treatment goals in T2DM, hypertension, COPD, CKD and CHD.

• Hands-on case management of individuals.

• Working in PHC team, with clear roles and responsibilities of team members.

• Engaging with participants’ families and using local resources to support effective self-management.

Participants in the control group will receive usual care. Data analysis and discussion will inform a revised intervention, which will then be provided to participants in the control (waitlist) group.

### Objective

We aim to compare the effectiveness of an integrated, family-centred and culturally safe model of care, compared with usual care, for improving tertiary preventive care for clusters of Indigenous people with complex chronic care needs.

### Outcomes

The primary outcome is reduction in HbA1c measured at 18 months. The secondary outcomes to be measured at the cluster level are:

• clinical care processes (initiation of self-monitoring of blood glucose, introduction of insulin for those with HbA1c>9%, appropriate use of ACEI/ARBs, aspirin, beta-blockers, statins, oral hypoglycaemic agents, agents for COPD and lung function monitoring)

• intermediate condition-specific outcomes (progression of estimated glomerular filtration rate, albumin creatinine ratio, blood pressure, lipids);

• avoidable hospitalisations;

• mortality;

• quality of life scores.

### Process evaluation

Factors relating to the delivery of the intervention will be documented and analysed. Key areas for evaluation include the:

• development and review of training materials and curriculum;

• interviews with IHWs and clients exploring the nature and continuity of the relationship between IHW and client; and

• extent of contact with clients and their families by IHWs.

### Sample size

The trial is powered to demonstrate a reduction in mean HbA1c by 1.0% over 18 months in the intervention group compared to the control group. This estimate is based on a mean drop of 1.3% HbA1c over one year (from 9.9% to 8.6%, following initiation of intensive drug treatment in T2DM) reported by a large US Health Maintenance Organisation [29]. A sample size of 49 in each group will have 90% power to detect a difference in mean HbA1c between the intervention and control group after 18 months of 1.0%, assuming that the common standard deviation is 1.5% using a two group t-test with a 0.050 two sided significance level. With 12 communities (six intervention, six control), this would require nine participants per community. However, due to the intervention being at the level of the health service and provider (cluster) rather than individual, the sample size needs to be inflated by the design effect, where:

$$\text{Design effect}=1+\left(\text{n}-1\right)\;\rho$$

where *n* is the average cluster number, and ρ the expected intra-class correlation coefficient for HbA1c. With n=9 and a ρ of 0.025 [22] the design effect = 1.2. Hence the required number of participants per community is 11 for the primary outcome. However, due to potential difficulty of maintaining participants in these communities in the trial, the potential for a more modest effect size in this group, and considering the relatively large number of secondary outcomes, we aim to recruit 30–35 participants in each community.

A second power calculation is based on expected reduction in avoidable hospitalisations in the intervention sites, related to the main chronic conditions. The estimated effect size is 0.08, based on the impact of the Torres Trial where an absolute reduction of 8% was achieved in the intervention sites over 12 months, and people with diabetes there were 40% less likely to be hospitalised with a diabetes-related complication compared to controls (RR=0.4) [9]. Thus, assuming a similar effect size, a two group Chi-square test with a 0.050 two-sided significance level will have 90% power to detect a difference in absolute reduction in avoidable hospitalisations of 8%, when the sample size in each arm is 125. Again, allowing for a design effect of 1.2, this would require 150 participants in each treatment arm.

#### Randomisation

Clusters were allocated to the intervention or waitlist groups using a simple randomisation method of pulling community names from a hat. Participants were enrolled by a local Indigenous worker nominated by the participating service. Allocation was concealed because clusters were randomly assigned to the intervention group after enrolment of participants. There is no masking of participants, IHWs or the research team.

#### Statistical methods

The primary statistical analysis will be by intention-to-treat, using generalised linear mixed effects models, taking into account clustering by community.

## Discussion

Improving Indigenous health and chronic disease management have been identified nationally and in Queensland as priority areas for investment by policy makers and funding agencies [30,31]. The rural and remote Indigenous health service delivery environment in north Queensland is characterised by high turnover of medical, nursing and allied health staff and specialist fly-in-fly-out services, hindering the systematic approach to service delivery required to effectively manage client care. Indigenous people often require additional support to access the health system, engagement with families to find effective solutions to health problems and better communication to understand the care or medications being prescribed to them [32]. To address these issues, efforts are required to enable, train, and encourage Indigenous people to take responsibility for programs and services that affect their health and for them to work closely with existing health-care systems [32].

This pragmatic trial will test a culturally-sound family-centred model of care with supported case management by IHWs to improve outcomes for people with complex chronic care needs. Its strong design ensures that the results will provide high quality evidence of the impact of such a model on meaningful outcomes. Other studies evaluating chronic care interventions have found that outcomes across communities can be variable [33]. Reports of pragmatic trials need to provide sufficient details about the setting, participants and intervention for users to determine if the results are generalisable to their own situation [34]. To this end, monitoring the fidelity of implementation to the model will be a key characteristic of the study [35].

This trial is now in the intervention phase. We expect to report on early lessons learnt from the planning and implementation of this trial shortly.

### Ethics committee approval

This protocol received approval from the Human Research Ethics Committees of University of South Australia, Cairns and Hinterland and the University of Queensland in November 2010.

## Abbreviations

ACEI: Angiotensin Converting Enzyme inhibitor; CHD: Coronary Heart Disease; CKD: Chronic Kidney Disease; COPD: Chronic Obstructive Pulmonary Disease; CVD: Cardiovascular Disease; FTE: Full time equivalent; ICST: Indigenous Clinical Support Team; IHW: Indigenous Health Worker; NT: Northern Territory; PHC: Primary Health Care; T2DM: Type 2 Diabetes Mellitus.

## Competing interests

The authors declare there are no competing interests.

## Authors’ contributions

BS has prepared the publication and is the key author of the discussion section of the article. RM developed the research design and project proposal from which the protocol is taken, undertook initial community consultation and obtained grant funding. AS has refined the protocol to ensure that it accurately reflects the project approach and implementation strategy and provided significant editorial support for the publication. CP participated in participant recruitment, provided input into the refinement of the project protocol and feedback to the draft article. ST led participant recruitment, provided input into the refinement of the project protocol and feedback to the draft article. MW lead the development of the family-centred model of care being implemented by Apunipima Cape York Health Council and incorporated into the research design, development of the project proposal and negotiation to establish the partnership with Apunipima Cape York Health Council. AE contributed to the development of the research design and project proposal. WH contributed to the development of the research design and project proposal. LS contributed to the development of the research design and project proposal. All authors read and approved the final manuscript.

## Pre-publication history

The pre-publication history for this paper can be accessed here:

http://www.biomedcentral.com/1471-2458/12/1017/prepub
